# Role of the Acidic Tail of High Mobility Group Protein B1 (HMGB1) in Protein Stability and DNA Bending

**DOI:** 10.1371/journal.pone.0079572

**Published:** 2013-11-08

**Authors:** Fabricio S. Belgrano, Isabel C. de Abreu da Silva, Francisco M. Bastos de Oliveira, Marcelo R. Fantappié, Ronaldo Mohana-Borges

**Affiliations:** 1 Laboratório de Genômica Estrutural, Instituto de Biofísica Carlos Chagas Filho, Universidade Federal do Rio de Janeiro, Rio de Janeiro, Brazil; 2 Laboratório de Helmintologia e Entomologia Molecular, Instituto de Bioquímica Médica, Universidade Federal do Rio de Janeiro, Rio de Janeiro, Brazil; 3 Weill Institute for Cell and Molecular Biology, Cornell University, Ithaca, New York, United States of America; CNR, Italy

## Abstract

High mobility group box (HMGB) proteins are abundant nonhistone proteins found in all eukaryotic nuclei and are capable of binding/bending DNA. The human HMGB1 is composed of two binding motifs, known as Boxes A and B, are L-shaped alpha-helix structures, followed by a random-coil acidic tail that consists of 30 Asp and Glu residues. This work aimed at evaluating the role of the acidic tail of human HMGB1 in protein stability and DNA interactions. For this purpose, we cloned, expressed and purified HMGB1 and its tailless form, HMGB1ΔC, in *E. coli* strain. Tryptophan fluorescence spectroscopy and circular dichroism (CD) experiments clearly showed an increase in protein stability promoted by the acidic tail under different conditions, such as the presence of the chemical denaturant guanidine hydrochloride (Gdn.HCl), high temperature and low pH. Folding intermediates found at low pH for both proteins were denatured only in the presence of chemical denaturant, thus showing a relatively high stability. The acidic tail did not alter the DNA-binding properties of the protein, although it enhanced the DNA bending capability from 76° (HMGB1ΔC) to 91° (HMGB1), as measured using the fluorescence resonance energy transfer technique. A model of DNA bending *in vivo* was proposed, which might help to explain the interaction of HMGB1 with DNA and other proteins, i.e., histones, and the role of that protein in chromatin remodeling.

## Introduction

**Figure 1:**
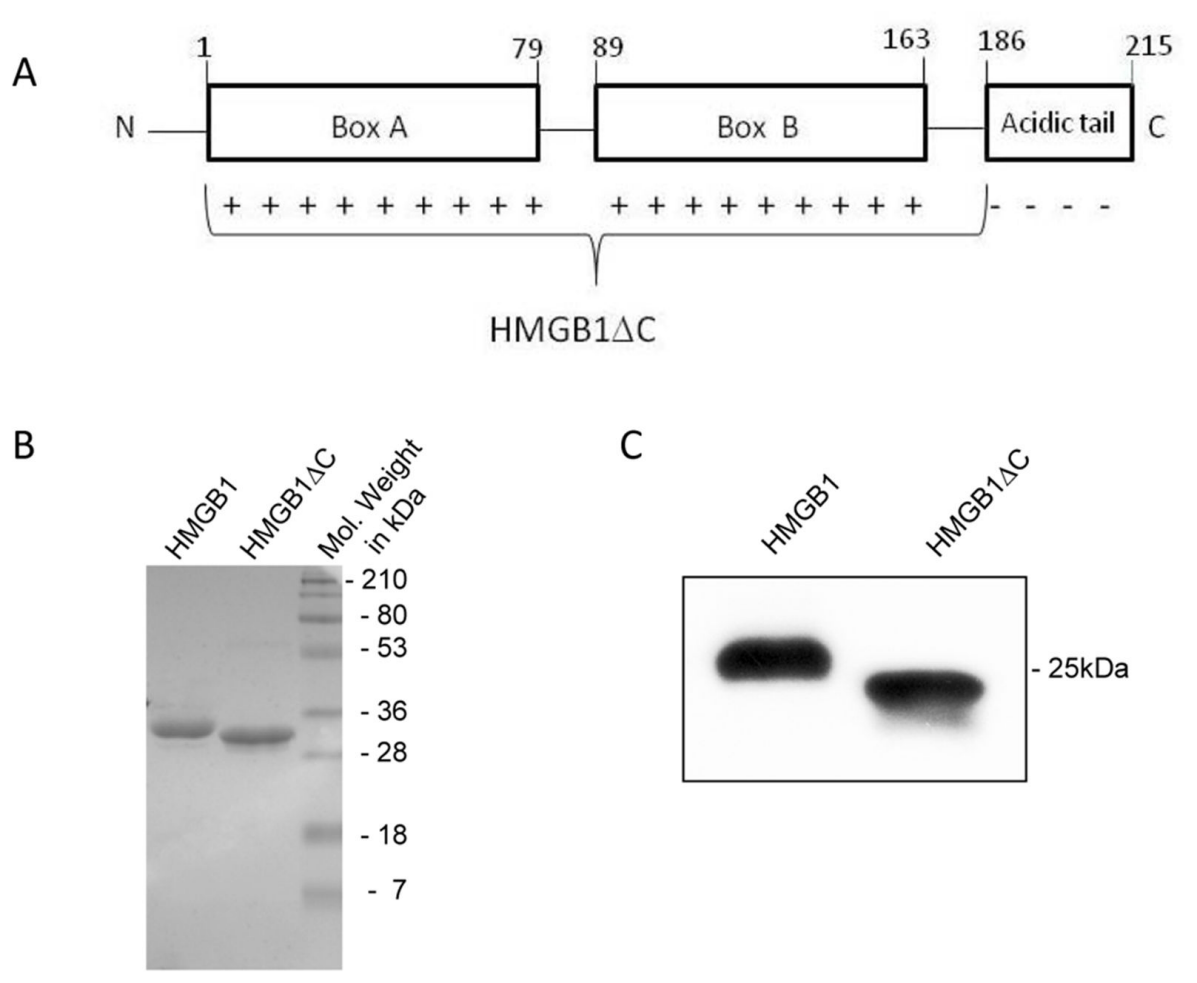
Structural organization of the human HMB1 protein. A) Schematic representation of the human HMGB1 structure showing Box A, Box B and the acidic tail motifs. Both boxes are rich in positive amino acid residues (+), whereas the acidic tail is exclusively composed of acidic amino acid residues (-) (residues 186-215). The removal of the acidic tail generated a truncated construct (HMGB1ΔC). B) Two micrograms of HMGB1 and HMGB1ΔC were separately applied onto a 15% SDS-PAGE. In a third lane, 5 μL the pre-stained molecular weight standards (Bio-Rad) were applied. The gel was stained by Coomassie Blue G-250 dye and. C) Western blotting with anti-human HMGB1 to confirm the recombinant protein identity. The 6His-Tag was not removed.

High mobility group box (HMGB) proteins belong to a superfamily of nuclear proteins with DNA-binding capabilities [1]. The human HMGB1 protein is composed of 215 amino acids and is functionally divided into three domains: two positively charged DNA-binding motifs (Boxes A and B) and a C-terminal domain composed of a segment of 30 acidic residues (Figure 1A). The two boxes are structurally similar, comprising 3 α-helices that confer an “L-shaped” DNA-binding domain, with an angle of 80° between the arms [2–5]. The minor groove of the DNA molecule binds to the concave side of the boxes with no sequence specificity. The current model of action suggests that the HMGB1 protein is capable of binding to and bending DNA randomly, remodeling chromatin in a “hit and run” fashion [6]. HMGB1 has been shown to have high affinity for topologically modified DNA, such as 4-way junctions and kinked, bulged and minicircle DNA [7–10].

HMGB1 proteins are extremely conserved in evolution, with 99% conservation in all mammalians studied, implying similar biological functions [11]. These proteins are also the most abundant non-histone protein in the nucleus, with one molecule per 10-15 nucleosomes [12]. The interaction with DNA is very dynamic and transient; HMGB1 was found to be the most mobile protein in the nucleus, crossing this organelle within 2 seconds [13,14]. 

The first DNA bending assay with HMGB1 was performed using the fluorescence resonance energy transfer (FRET) technique using the protein from *Chironomus* [15]. These experiments revealed that HMGB1 could promote a bending angle of 150°. Subsequently, another study measured the bending angle of HMG-D and HMG-Z from *Drosophila*, cHMG1a of *Chironomus* and NHP6A from *Saccharomyces cerevisiae* [16]. The protein lacking the C-terminal acidic tail (HMGB1ΔC) or one of the boxes was studied by atomic force microscopy (AFM) and dual-laser beam optical tweezers [17,18]. The two techniques determined similar bending angles, with 67° for HMGB1ΔC and 77° for boxes A or B.

The acidic tail of HMGB1 is an important modulator of its DNA-binding properties [19,20]. Several reports showed that the this tail lowers the DNA affinity and supercoiling activity [21,22]. The short tail (12 residues) from HMG-D of *Drosophila* appears to have an affinity for certain structures because it binds to 4-way junction DNA and cisplatin-modified DNA but not to DNA minicircles [23]. The acidic tail may interact with other proteins, such as histones H1 and H3 [24,25].

Although HMGB1 proteins have been the focus of intensive structural and functional studies, an investigation of the role of the acidic tail of human HMGB1 in protein stability and DNA bending is still lacking. In this work, we aim at evaluating the thermodynamic stability promoted by the interaction between the boxes and the acidic tail of HMGB1. In addition, we describe an investigation of the relationship between the structure of the acidic tail and the DNA bending activity of HMGB1 in solution. 

## Results

### The acidic tail and protein stability of the human HMGB1

To investigate the role of the human HMGB1 acidic tail in protein stability and DNA bending, the full-length protein and its tailless form (HMGB1ΔC) were expressed and purified. A schematic representation of boxes A and B and the acidic tail is shown Figure 1A. The purity and identity of HMGB1 and HMGB1ΔC were confirmed by 15% SDS-PAGE (Figure 1B) and by western blotting using monoclonal antibody anti-human HMGB1 (Figure 1C), respectively. 

**Figure 2:**
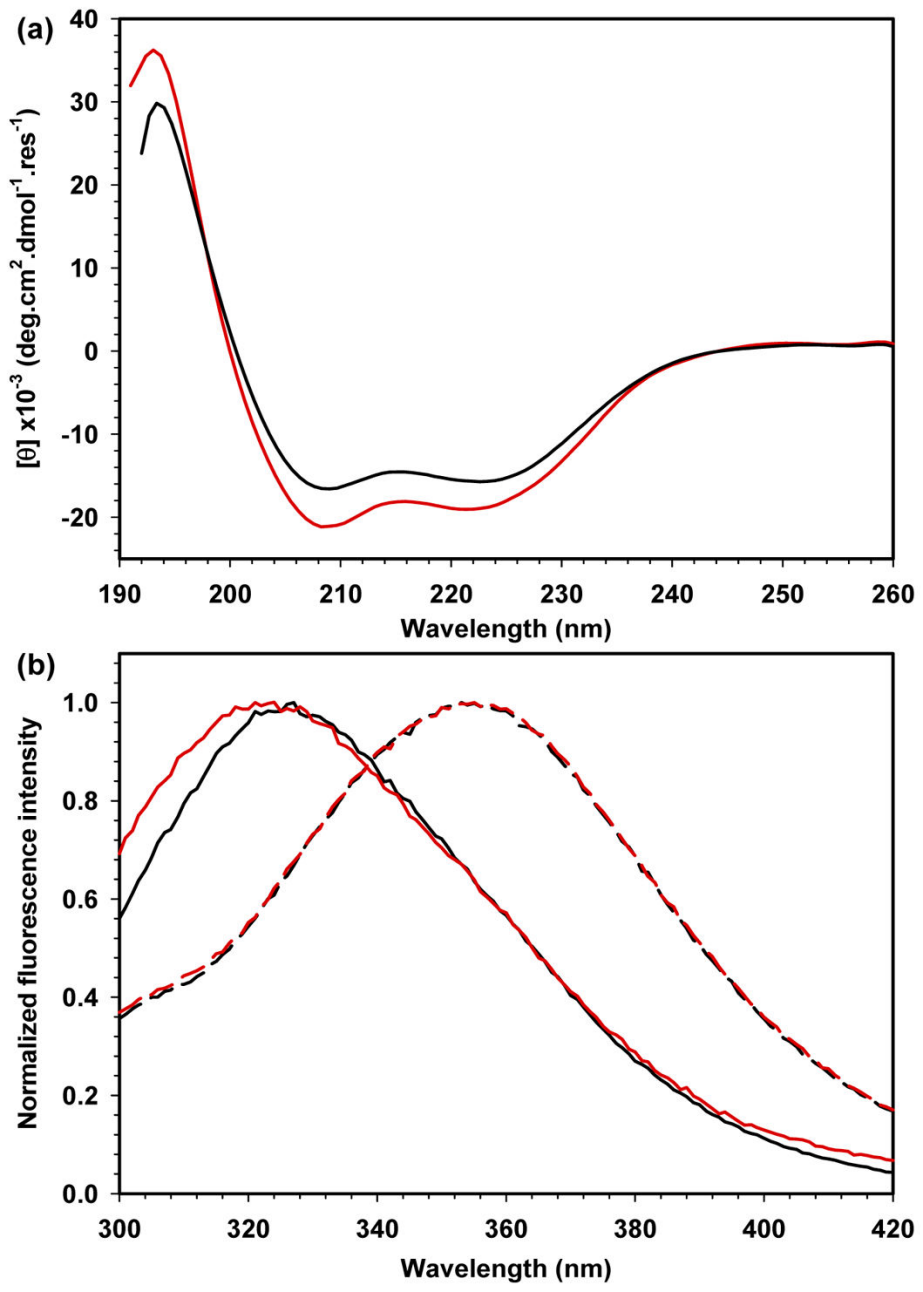
Analysis of the secondary and tertiary contents of HMGB1 and HMGB1ΔC by CD and Trp fluorescence spectroscopies. A) CD spectra of 5 μM HMGB1 (black lines) and HMGB1ΔC (red lines) at 25 °C and neutral pH. Each spectrum was converted to molar ellipticity for proper comparison. B) Normalized Trp fluorescence spectra of 5 μM HMGB1 and HMGB1ΔC in the native state (straight lines) and denatured state with 5.5 M Gdn.HCl (medium-dashed lines). All experiments were performed at 25 °C, and the buffer composition was 10 mM Tris.HCl at pH 7.2, 50 mM NaCl, 0.5 mM DTT, 0.1 mM EDTA and 5% glycerol.

The secondary and tertiary structures of HMGB1 and HMGB1ΔC were monitored by circular dichroism (CD) and Trp fluorescence spectroscopy, respectively, to assess whether the proteins were properly folded during the purification steps and to determine the effect of the acidic tail on HMGB1-folding. As expected, both the HMGB1 and HMGB1ΔC proteins revealed basically α-helical structures, with negative peaks at 208 and 222 nm (Figure 2A). However, the molar ellipticity signal for HMGB1 was less negative, suggesting a slightly higher content of random coil conformation because of the acidic tail, which is known to be highly disordered [26,27]. In addition, the fluorescence spectroscopy analysis of the Trp residues 49 and 133 (located in Boxes A and B, respectively) showed that the maximum fluorescence intensity of approximately 325 nm was observed in both the HMGB1 and HMGB1ΔC spectra (Figure 2B, solid lines). When both proteins were incubated in 5.5 M guanidine hydrochloride (Gdn.HCl), a significant red shift of their spectra to higher wavelengths (peaks at approximately 360 nm) was observed, which is characteristic of a complete exposure of the Trp residues to the milieu (Figure 2B, medium dashed lines). Altogether, these results confirm that both HMGB1 and its tailless construct were obtained in folded conformation after the purification processes and suggest that the acidic tail does not apparently affect the final folded conformational state of boxes A and B. 

**Figure 3:**
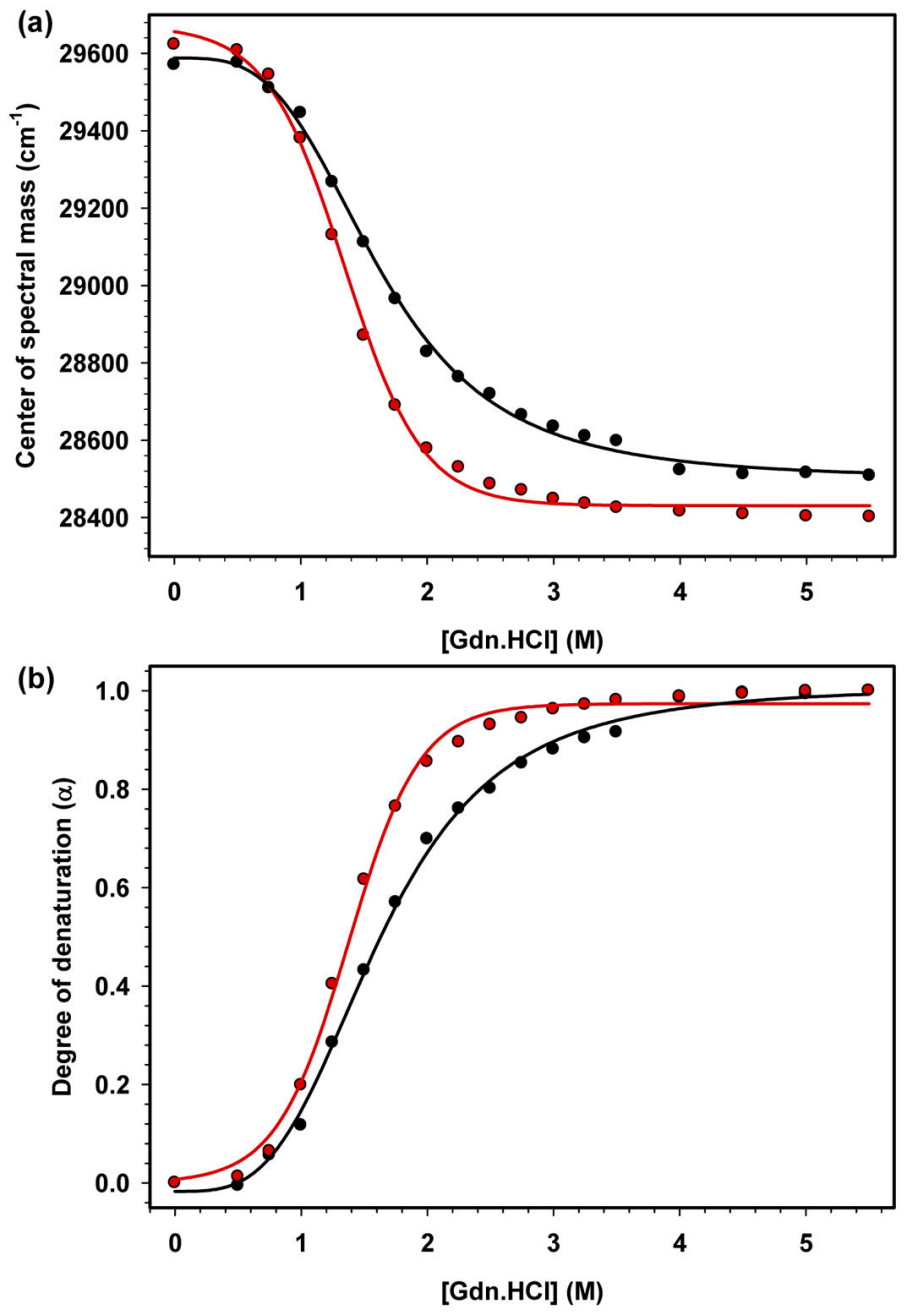
Denaturation of HMGB1 and HMGB1ΔC as a function of increasing Gdn.HCl concentration. A) The CM of HMGB1 (black circles) and HMGB1ΔC (red circles) at 5 μM was obtained for each [Gdn.HCl] using Equation 1, as described in the Material and Methods Section. B) Trp fluorescence spectra were obtained and converted to degree of denaturation (α) according to Equation 2. The resistance to unfolding can be analyzed by G_1/2_, which reflects the concentration necessary to unfold 50% of the protein population and is detailed in Table 1.

To evaluate the effect of the acidic tail on HMGB1 stability, both the full-length and the tailless proteins were subjected to increasing concentration of Gdn.HCl from 0 to 5.5 M, and protein denaturation was monitored by a red shift in their Trp fluorescence spectra. A decrease of the center of spectral mass (CM) (calculated from Equation 1) from approximately 29,600 to 28,500 cm^-1^ was obtained from the denaturation curves for both proteins (Figure 3A). The CM values were then converted into degree of denaturation (α) according to Equation 2, and the curves were fitted as previously described (Figure 3B) [28,29]. The Gdn.HCl concentration required to obtain 50% protein denaturation (G_1/2_) of HMGB1 and HMGB1ΔC was 1.6 and 1.3 M, respectively (Figure 3B), whereas the calculated free Gibbs energy (ΔG_H2O_) was 2.4 and 1.7 kcal/mol, respectively (Table 1). These results indicate that HMGB1ΔC is less stable against Gdn.HCl denaturation than HMGB1. Similar results were obtained for urea denaturation (data not shown), implying an important role of the acidic tail for the increased thermodynamic stability of the HMGB1 structure, most likely as a consequence of the interactions between the boxes and the acidic tail [30].

**Table 1:** Thermodynamic parameters for HMGB1 and HMGB1ΔC proteins.

Protein	T_m_ (°C)*	G_1/2_ (M)	*m* Gdn.HCl (kcal/mol.M)	ΔG_H2O_ (kcal/mol)
HMGB1	48.6 ± 0.2	1.62 ± 0.02	1.9 ± 0.2	2.4 ± 0.2
HMGB1ΔC	43.2 ± 0.2	1.34 ± 0.02	1.3 ± 0.1	1.7 ± 0.2

* These values were obtained from the thermal denaturation monitored by Trp fluorescence spectra. The values obtained from the CD curves are the same and thus were not included in the table.

**Figure 4:**
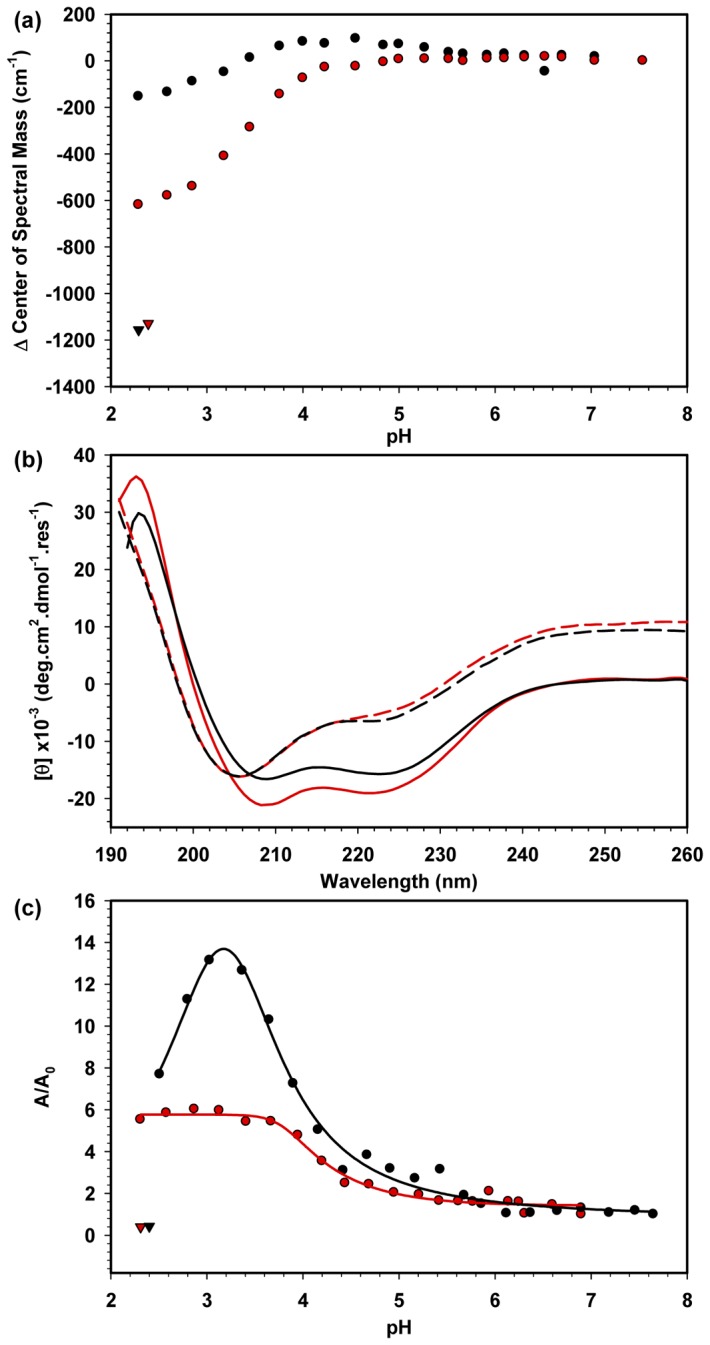
Influence of low pH on the HMGB1 structure. A) HMGB1 (black circles) and HMGB1ΔC (red circles) at 5 μM concentration were incubated at different pH values (in citrate/citric acid buffer), and the CM variation (ΔCM) was calculated. Because of the small change in ΔCM, even in a very acidic pH, both proteins were also incubated with Gdn.HCl at pH 2.3 and 5.5 M (black triangle for HMGB1 and red triangle for HMGB1ΔC). B) The secondary structure content of 5 μM HMGB1 at neutral pH (black straight lines) and pH 2.3 (black medium-dashed lines) and of HMGB1ΔC at neutral pH (red straight lines) and pH 2.3 (red medium-dashed lines) was monitored by CD at 20 °C. Spectra were converted to molar ellipticity, as described in the Material & Methods section. C) The interaction of bis-ANS and the proteins was assessed by exciting 10 μM probe in a solution containing 5 μM HMGB1 (black circles) or HMGB1ΔC (red circles) at different pH values after a 1-h incubation at 25 °C. For comparison, HMGB1 and HMGB1ΔC were incubated at pH 2.3 in the presence of 5.5 M Gdn.HCl (closed triangles). Normalized spectrum areas were obtained by dividing the spectrum area value of each pH point by the area value at neutral pH.

The role of electrostatic interactions between the acidic tail and the HMG box domains and the effect of these interactions on the thermodynamic stability of HMGB1 were further evaluated at low pH (from 7.5 to 2.3) by the CD and Trp fluorescence spectra of HMGB1 and HMGB1ΔC. Both proteins were partially denatured as the pH decreased, but significant tertiary and secondary structure was still detected (Figures 4A and 4B). The decrease in the CM between pH 7.5 and 2.3 for HMGB1 and HMGB1∆C was 200 and 600 cm^-1^, respectively (Figure 4A), and this decrease was observed only at pH values lower than 4.5, suggesting that both proteins were stable at mildly acidic conditions (pH above 4.5). This CM variation was considerably smaller than that obtained in the Gdn.HCl denaturation curves (~ 1100 cm^-1^) (Figure 3A), mainly for HMGB1, whose tertiary structure was shown to be very resistant to denaturation at low pH. In addition, significant residual α-helix content was observed for both proteins when their secondary structure was monitored by CD under very acidic conditions (pH 2.3) (Figure 4B). These results demonstrated again that the acidic tail plays an important role in the structural stability of the HMGB1 protein. The stabilization promoted by the Asp and Glu residues in the acidic tail was also evident when the fluorescent probe bis-ANS was used to monitor the denaturation of HMGB1 at low pH (Figure 4C). The fluorescence emission of bis-ANS that was free in solution was almost undetectable, but it increased significantly as bis-ANS bound non-covalently to the hydrophobic core/clusters usually present in partly folded proteins; therefore, this probe is often used to monitor protein denaturation [31]. A significant 14-fold increase in the area ratio of the bis-ANS spectra (A/A_0_) upon interaction with HMGB1 was observed at pH 3.5 relative to the spectral area obtained at pH 7.5 (A_0_); this change decreased to 8-fold as the pH was further lowered to 2.3, clearly indicating the formation of the hydrophobic clusters typically found in partly folded proteins. Conversely, the increased A/A_0_ observed for HMGB1ΔC at this same pH range was much less pronounced (6-fold increase), also indicating the formation of such clusters; however, the HMGB1ΔC structure appears to be more unfolded than the full-length protein. The bis-ANS fluorescence was only abolished when both proteins were incubated at pH 2.3 in the presence of 5.5 M Gdn.HCl (Figure 4C, closed triangles). Therefore, while the secondary structure content of both proteins was slightly disturbed when subjected to low pH, their tertiary structure was significantly affected, generating hydrophobic cavities detected by bis-ANS probe, especially for HMGB1 (Figure 4C). These results also confirmed that the presence of the acidic tail increased the structural stability of the HMGB1 protein, most likely due to its interactions with the HMG boxes, as shown previously [27]. 

**Figure 5:**
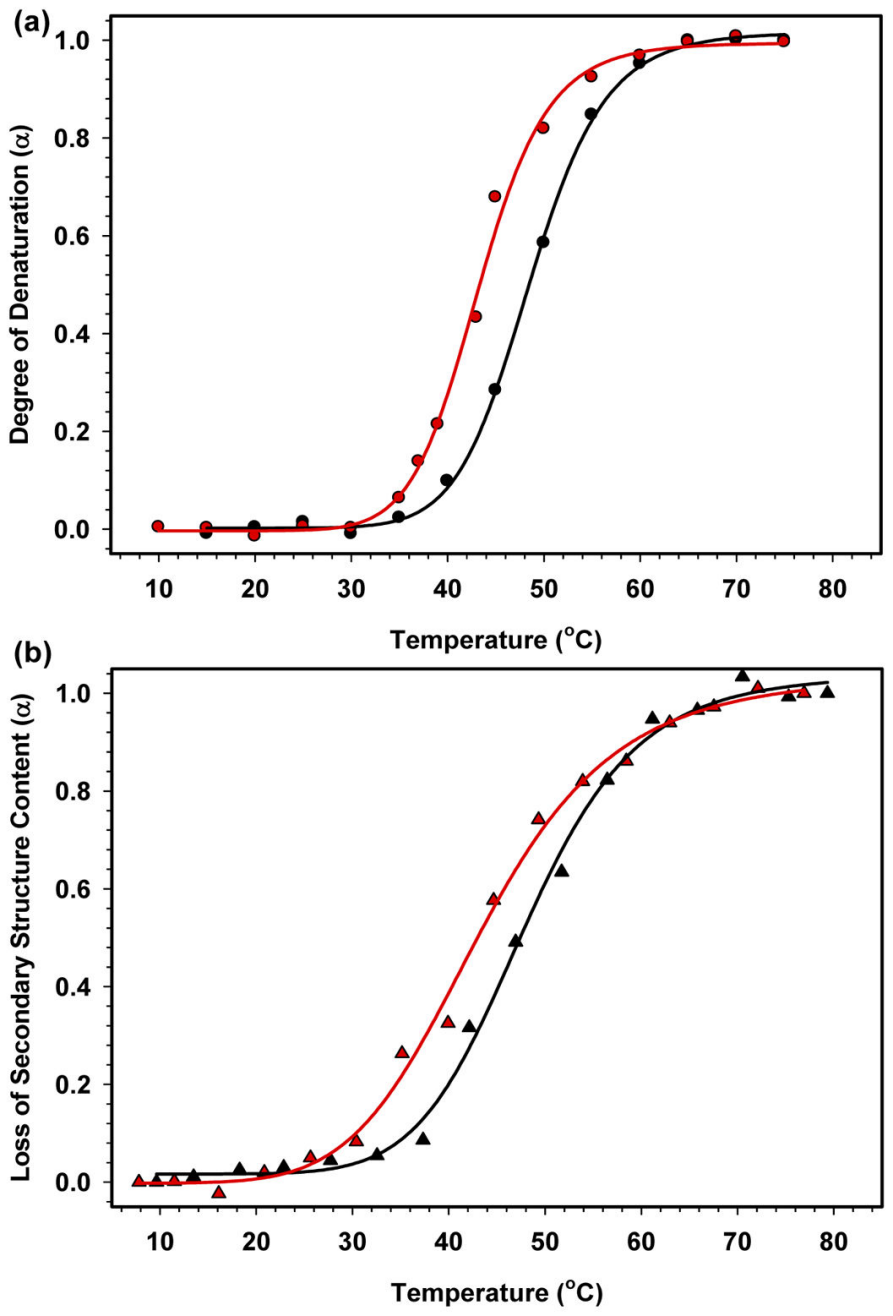
Thermal denaturation of the HMGB1 protein. A) The Trp fluorescence emission spectra of HMGB1 (black circles) and HMGB1ΔC (red circles) at each temperature were acquired and converted into CM and α according to Equations 1 and 2, respectively. The curves were adjusted by sigmoidal fitting, and the T_m_ was obtained directly from the fitting. B) The CD signal at 222 nm for the HMGB1 and HMGB1ΔC spectra at each temperature was converted into the loss of secondary structure content. The buffer contained 10 mM Tris.HCl at pH 7.2, 50 mM NaCl, 0.5 mM DTT, 0.1 mM EDTA and 5% of glycerol.

The thermal stability of HMGB1 and HMGB1ΔC was also monitored using Trp fluorescence and CD spectroscopies. When the two proteins were subjected to a temperature change between 5 and 75 °C (in the fluorescence experiment) and between 10 and 80 °C (in the CD experiment), HMGB1 clearly demonstrated higher thermostability than the tailless construct, as reflected by their melting temperature in both Trp fluorescence (48.6 °C for HMGB1 and 43.2 °C for HMGB1∆C) and CD (48.0 °C for HMGB1 and 43.4 °C for HMGB1∆C) experiments (Figure 5 and Table 1). The thermal denaturation process of both proteins was fully reversible (data not shown). Once again, the presence of the acidic tail increased the thermal stability of the HMGB1 protein, as previously observed in other studies [26,27,32]. Moreover, the thermal denaturation curves strongly suggested that both the full-length and acidic tailless proteins lost both secondary and tertiary structures in a concerted manner, as observed from the superposition of their respective Trp fluorescence and CD curves.

### Protein-DNA interactions

**Figure 6:**
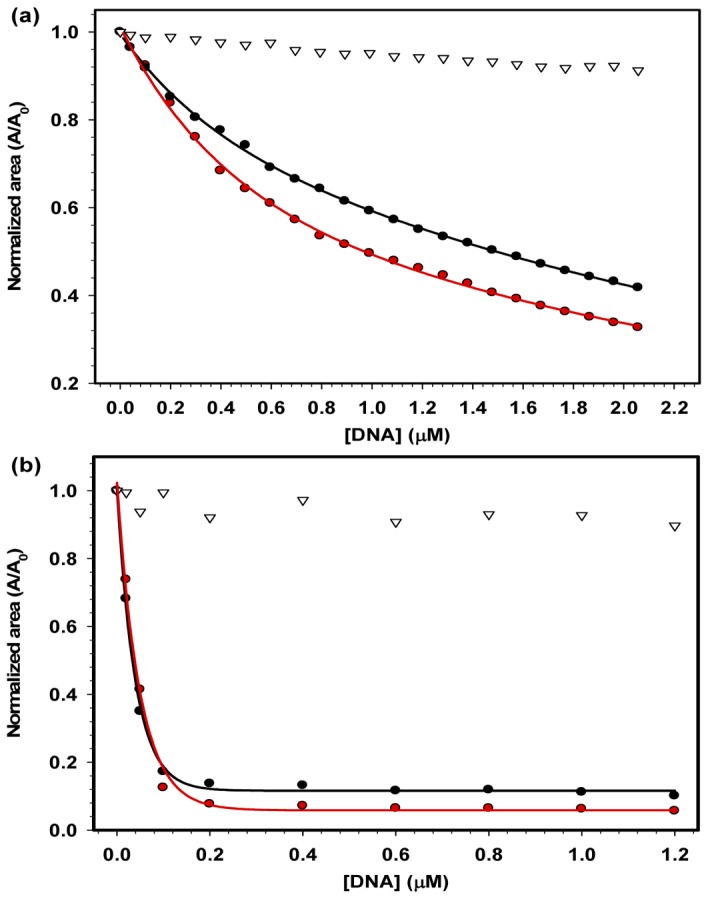
Binding of HMGB1 protein to linear dsDNA monitored by fluorescence spectroscopy. A) Interaction between HMGB1 (black circles) or HMGB1ΔC (red circles) with 20-bp DNA was analyzed by the quenching of the Trp emission fluorescence. Both proteins were kept at 2 μM, and the DNA concentration was varied from 0 to 2 μM. Trp emission spectra were collected after a 15-min incubation at 25 °C. B) Interaction between HMGB1 or HMGB1ΔC with 20-bp DNA, as analyzed by bis-ANS displacement. The protein and bis-ANS concentrations were 0.5 μM and 10 μM, respectively, whereas the DNA concentration varied from 0 to 1.2 μM. The emission spectra of bis-ANS were acquired after a 15-min incubation time at 25 °C. Normalized spectrum areas were calculated as described in Figure 4. Control experiments were performed similarly but in the absence of protein.

 The interactions between DNA and HMGB1 of several different species have previously been studied using non-equilibrium methods, such as gel-shift retardation assays [33,34], which are not accurate techniques for measuring binding constants [35]. To measure accurately the binding constants between HMGB1 and DNA molecules at equilibrium, different spectroscopic techniques have been employed. Interestingly, DNA molecules can quench the fluorescence of the Trp residues present in the HMGB1 sequence, indicating that protein-DNA interaction could be monitored by Trp quenching experiments; thus, the effect of the acidic tail on this interaction could be studied (Figure 6A). As the DNA concentration increased, the fluorescence quenching became slightly higher for HMGB1∆C than for HMGB1 but significantly higher than for the control curve (open triangle). This result indicated a stronger binding of the tailless construct to DNA. To confirm these results, the bis-ANS probe was also used to monitor protein-DNA binding. The increase in DNA concentration promptly displaced bis-ANS that was bound to the hydrophobic core of HMGB1 and HMGB1ΔC proteins (Figure 6B). Both the Trp and bis-ANS quenching approaches demonstrated that the acidic tail did not interfere with binding of the HMG boxes to linear DNA.

**Figure 7:**
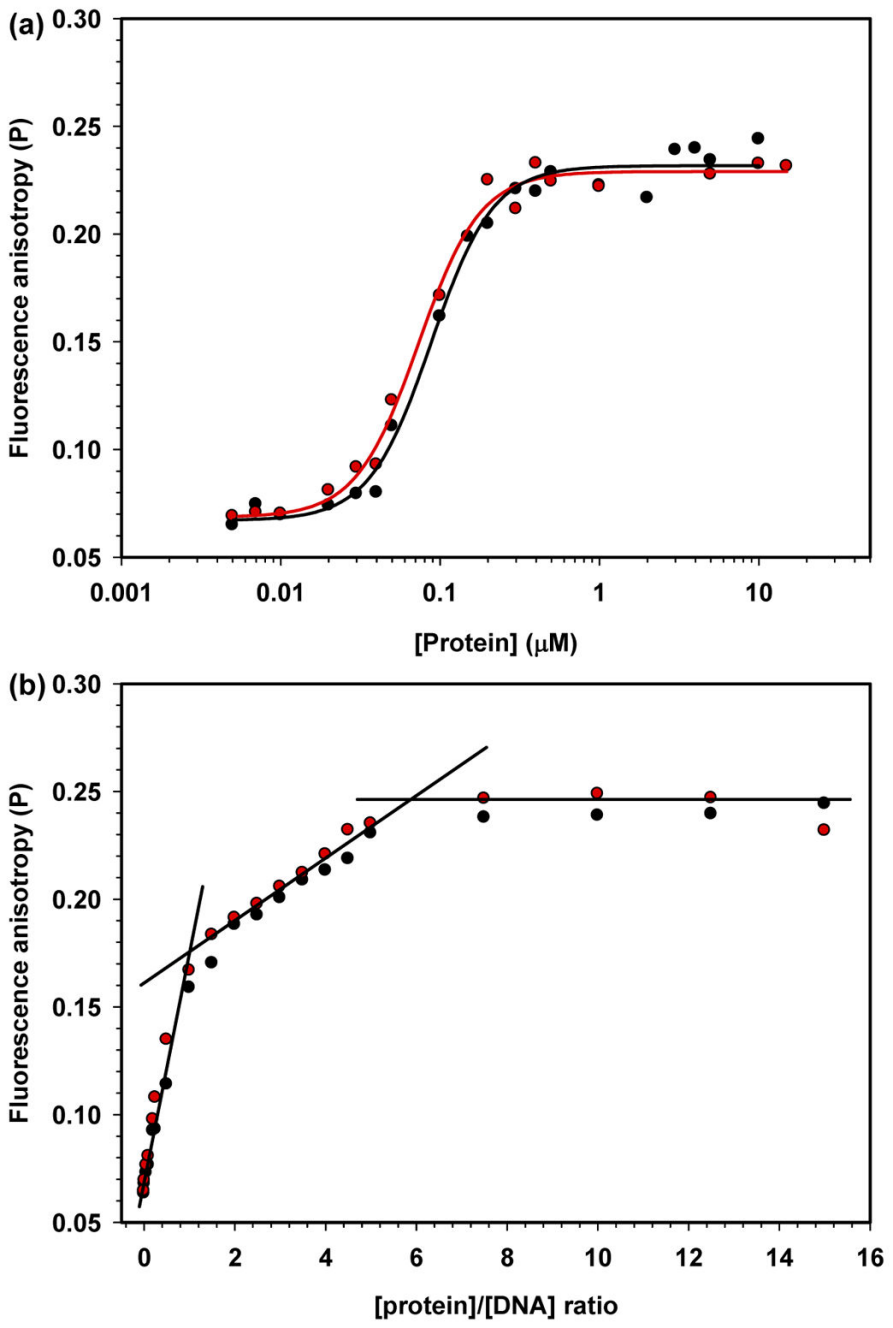
Binding isotherm of HMGB1 to fluorescently labeled linear DNA. A) FAM-labeled 20-bp dsDNA at a 50 nM concentration was titrated with increasing HMGB1 (black circles) or HMGB1ΔC (red circles) concentrations, and the fluorescence polarization (P) of the fluorescent probe was measured after a 15-min incubation at 25 °C. (a) The binding stoichiometry of HMGB1 or HMGB1ΔC to FAM-labeled dsDNA was calculated. Increasing protein concentrations were added to a solution containing a mixture of 2 μM unlabeled dsDNA and 50 nM FAM-labeled dsDNA; thus, the [Protein]/[DNA] ratio varied from 0 to 15. The polarization values were measured by exciting the probe at 490 nm and reading the FAM-emission fluorescence at 520 nm after a 15-min incubation at 25 °C.

To measure the binding constants for both proteins, fluorescence anisotropy studies using 20-bp DNA were also performed; the DNA was labeled with carboxyfluorescein (6-FAM) at the 5'-end of one of the DNA strands. DNA binding isotherms for HMGB1 and HMGB1∆C were generated by monitoring the increase in the fluorescence anisotropy of the labeled DNA molecules; the fluorescence anisotropy increased because of the formation of the protein-DNA complex upon the addition of increasing protein concentrations [36]. The DNA binding constants for HMGB1 and HMGB1∆C were very similar (K_d_ = 88 ± 5 and 72 ± 4 nM, respectively), indicating that the HMG boxes are the domains responsible for DNA-binding affinity, i.e., the acidic tail does not significantly influence the HMGB1 interaction with short, linear DNAs (Figure 7A). The stoichiometry ratio of the interaction was assessed using anisotropy studies with different protein-DNA ratios. The strategy of this experiment was based on the continuous binding of protein molecules to the DNA template up to the point in which all available binding sites were saturated and the anisotropy signal reached a plateau. The fluorescence anisotropy increased linearly until a 1:1 [protein]/[DNA] ratio was achieved, indicating that all available DNA-probes were bound (Figure 7B). Curiously, as the protein concentration was further increased above a [protein]/[DNA] ratio of 5:1, another plateau was reached, suggesting that additional HMGB1 molecules interacted with each other to form a larger aggregated complex. This finding could be explained by the fact that the acidic tail of a molecule could form inter-molecular interactions with the HMG boxes of another molecule. Altogether, our data confirmed previous results obtained with calf HMGB1, in which both proteins presented the same HMGB1-DNA ratio of 1:1 and that the presence of the acidic tail had no effect on the protein-DNA interaction [37].

Although there are some studies measuring DNA bending by HMGB1, none of them compared the full-length and truncated proteins [16,17,38]. In this work, 20-bp DNA molecules labeled with FAM, TAMRA, or FAM and TAMRA were used to calculate the bending angle promoted by both proteins using the fluorescence resonance energy transfer (FRET) technique. FRET is the radiationless transfer of energy from an excited donor fluorophore (FAM) to a suitable acceptor fluorophore (TAMRA) [39]. The excitation spectrum of the acceptor must partially overlap with the fluorescence emission spectrum of the donor for FRET to occur. The FRET efficiency depends on the distance between the two fluorophores. Therefore, the greater the nucleic acid bending angle is, the closer is the distance between the two fluorophores and thus, higher is the FRET efficiency (see Material & Methods). The FRET efficiency (FE) was obtained after making all adjustments and corrections for possible probes or protein interference in the fluorescence data. An FE value of 0.33 was obtained for HMGB1, while a smaller value of 0.23 was calculated for HMGB1ΔC. Comparing these to the value of 0.10 obtained for free DNA provides the first indication that the DNA bending occurred. The higher value for full-length protein indicated the closer proximity of the probes. HMGB1 was able to increase the proximity of the two probes by bending the DNA to a distance of 56 Å. This distance is considerably less than the distance of 61 Å obtained for HMGB1ΔC; consequently, the FRET efficiency for HMGB1 was considerably higher than that for HMGB1ΔC. A model of DNA bending is necessary to estimate the bending angle from the distance between the probes [38]. The two-kinked model is typically used to study human proteins with HMG-box motifs and was, therefore, used in this study [40,41]. Table 2 summarizes these parameters and clearly shows the greater bending capacity of HMGB1 when compared with that of HMGB1ΔC. The bending angle for HMGB1 was 91°, in contrast to 76°, which was obtained for the tailless construct.

**Table 2:** Parameters of DNA bending promoted by HMGB1 protein obtained by FRET.

	DNA	DNA+HMGB1	DNA+HMGB1ΔC
FRET efficiency (FE)	0.10 ± 0.04	0.33 ± 0.05	0.23 ± 0.03
Distance between probes (Å)	73 ± 6	56 ± 2	61 ± 2
Bending angle (°)	n.a	91 ± 7	76 ± 7

## Discussion

The recent increase in HMGB1 studies may be attributed to its role in many diseases, ranging from viral infections to autoimmune disorders and cancer [42–44]. The C-terminal acidic tail of HMGB1 appears to play a crucial role in the maintenance of protein stability and, consequently, its proper function. In the present study, we aimed at understanding the structural and functional relationship between the acidic tail and the HMG boxes of the full-length HMGB1 and the effect of this tail on DNA binding and bending. Furthermore, as far as we know, this report is the first that analyzes the differences in protein stability and DNA bending between the human HMGB1 and its tail-less construct. We showed that the acidic tail does not significantly affect the secondary structure of HMGB1, corroborating previous reports [26]. Nonetheless, the absence of the acidic tail destabilizes the tertiary structure of HMGB1, favoring its denaturation (this work and Elenkov et al. 2011) [26].

The denaturation curves clearly showed the role of the acidic tail in the thermodynamic stability increase of the HMGB1 protein, which was reflected in a higher ΔG_H2O_ [29]. The *m* is directly proportional to the solvent-accessible surface area (ΔASA), and the higher value for the full-length protein was expected because it has more amino acid residues [45]. The *m* values obtained with urea were approximately half those of Gdn.HCl (data not shown), which is typically found in many proteins and reflects the greater denaturant strength of Gdn.HCl [45].

Thermal unfolding strengthens the importance of the acidic tail in protein integrity. This work clearly demonstrates a steep shift from the folded to the unfolded state for HMGB1∆C between 40 and 50 °C, in agreement with previous reports [27]. Thomas and colleagues obtained comparable T_m_ results for HMGB1 and HMGB1∆C (50 °C and 44 °C, respectively). Interestingly, high hydrostatic pressure experiments have shown that both proteins are in a monomeric state and that thermal unfolding occurs in a very similar manner (data not shown). These results suggest that intra-molecular interactions between the boxes and the acidic tail, rather than intermolecular interactions, are responsible for the protein stabilization. NMR analyses have shown specific interactions of the acidic tail with both boxes, regardless of the acidic nature of the tail and the basic nature of the boxes [27]. 

Because the interaction between HMG boxes and the acidic tail is mainly electrostatic, it would be affected by solution pH. An acidic environment promotes changes in the charges of amino acid residues, generating electrostatic repulsions that lead to protein denaturation [46]. Low pH partially disturbed the secondary structure of the full-length HMGB1 and HMGB1ΔC. In contrast, the tertiary structure of the truncated version was more affected by the low pH, most likely because the acidic (negative) tail in the full-length protein compensates the high density of positive charges in the HMG boxes. This finding was also reflected in the presence of a more prominent folding intermediate state at low pH for HMGB1, revealed by bis-ANS fluorescence.

We have also characterized the binding of HMGB1 to short DNA stretches in solution using fluorescence techniques, such as fluorescence anisotropy and FRET. We chose a 20-bp B-DNA substrate to promote protein-DNA binding in a 1:1 ratio, as previously reported [16,47]. Protein-DNA interaction induces Trp quenching, which makes this amino acid residue a good probe for binding monitoring [35], especially for HMGB1 because both Trp residues are very close to the intercalating residues Phe 37 and Ile 121 [48]. Both Trp quenching and bis-ANS displacement demonstrated a similar binding affinity for the linear DNA sequence, further indicating that the acidic tail does not significantly affect the binding affinity of HMGB1 for DNA but acts as a regulator of the protein-DNA interaction [23,49].

To evaluate the binding affinity of HMGB1 and HMGB1∆C, fluorescence anisotropy was measured using a fluorescent-labeled DNA sequence. The binding isotherms clearly demonstrated a similar binding affinity of approximately 80 nM, corroborating the large binding affinity for modified DNA, such as hemicatenated DNA loops (K_d_ < 0.2 x 10^-12^ M), minicircles (1 x 10^-10^ M) and 4-way junctions (1 x 10^-9^ M) [8–10,19]. The binding stoichiometry for the 20-bp linear DNA suggested a 1:1 ratio, and the acidic tail appears to have no influence on this parameter, as previously shown for HMGB1 and HMGB1∆C from calf thymus [37].

Although there are many reports in the literature characterizing the binding or bending of HMGB1 to discrete structured DNA motifs [7–10], the binding features of human HMGB1 to linear duplex DNA in solution have been poorly characterized [33,34]. Using the energy transfer between donor-acceptor probes attached to the two 5' ends of linear DNA, the bending angle of the nucleic acid could be measured. The FRET efficiency promoted by the full-length HMGB1 was considerably higher than for HMGB1∆C, corresponding to a distance between the probes of 56.4 and 60.9 Å, respectively. The two-kinked model of bending, which is typically used for HMG-box proteins [40,41,50], was used to estimate the bending angle from the FE values. This model is based on a crystal structure of TBP binding to TATA box DNA [51], which represents the DNA molecule as a rod with three sections with lengths R1, R2 and R3. DNA bending generates two "hinges" between R1-R2 and R2-R3. Other groups have successfully used the two-kinked model even though it does not account for unwinding/twisting of DNA molecule upon bending [40,41]. The two-kinked model generates intermediate bending angles when compared to single central (higher bending angle) and continuous smooth bending models (lower bending angle) [50]. In principle, the possibility of DNA twisting during TBP-induced DNA bending was then proposed to improve the two-kinked model [41], contributing to the end-to-end distance between the FRET probes. However, the twisting may cause a tension increase within the DNA strands, making this model energetically less favorable than simple bending. Moreover, different combinations of twisting can achieve the same bending angle. Therefore, the induction of DNA twisting upon the HMGB1 protein binding might only be confirmed experimentally from the structure determination of the protein-DNA complex using high-resolution techniques (i.e. X-ray crystallography and NMR).

The first bending angle calculated from non-specific linear DNA in solution was for *Chironomus* HMGB1 [15]. A bending angle of 150° was initially obtained, but soon after, Lorenz and colleagues obtained a smaller value of 95° for this same protein [16]. This work also evaluated the bending angle of ortholog HMGB proteins from *Drosophila* and *Saccharomyces cerevisiae* and their tailless constructs. In these cases, there was no difference in the DNA bending among these different proteins, which could be explained by their short acidic tail (approximately 12 amino acid vs 30 for human HMGB1).

Curiously, the application of two-kinked model showed that the presence of the acidic tail led to a 20% increase in the DNA bending angle; we calculated bending angle values of 91° for HMGB1 and 76° for HMGB1∆C, which are in agreement with the value obtained for many other HMG box-containing proteins, such as TBP (80°), SRY (83°), IHF (80° per monomer), NHP6A (70°) and HMGB2 Box A (87°) [38,52–55]. These similar values might indicate a steric hindrance for DNA bending by this protein motif. While no bending angle calculated for human full-length HMGB1 has been published, the HMGB1∆C bending angle has been calculated using several techniques. Measurements using the atomic force microscopy (AFM) and dual-laser beam optical tweezers techniques revealed bending angles of 67° and 77°, respectively [17,18], which are in excellent agreement with the value calculated for HMGB1∆C protein in our study.

This work was the first to demonstrate a 15° (or 20%) increase in DNA bending promoted by the acidic tail in human HMGB1, and this augment might have important biological functions. It was previously demonstrated that HMGB1∆C is not capable of inducing transcript stimulation nor can it participate in chromatin remodeling [24,56,57]. Our work might shed light on those experiments, suggesting that an increase in bending capacity (but not binding affinity) promoted by the acidic tail may be an important factor responsible for this phenomenon.

**Figure 8:**
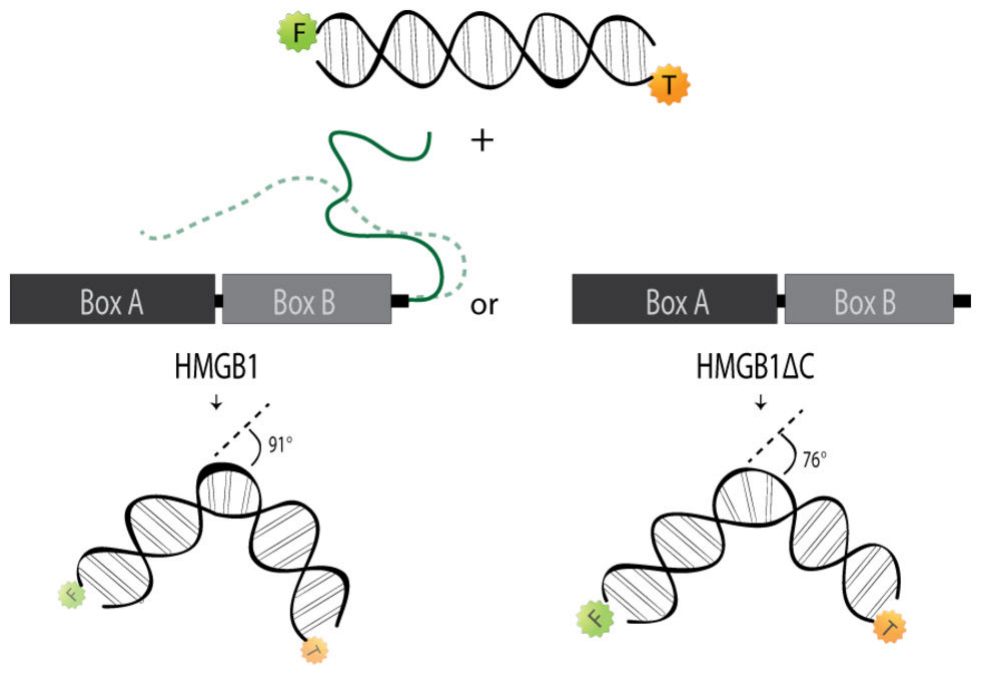
Schematic representation of HMGB1-mediated DNA bending. A 20-bp oligonucleotide labeled with FAM (green star, F) and TAMRA (orange star, T) fluorophores in the presence of HMGB1 or HMGB1∆C undergoes bending at different angles, measured by the distance between these two fluorophores. Bending angle values were obtained using the two-kinked model. The difference observed in size and color intensity of the fluorophores molecules is proportional to their emission quenching. The acidic tail of HMGB1 and its interaction with other part of the molecule are represented by green and dashed lines, respectively.

We have proposed a model of the HMGB1-DNA bending interaction to try to explain the role of the acidic tail in “boosting” DNA bending (Figure 8). NMR studies previously demonstrated that this tail has extensive contacts with HMG boxes, restricting the tail conformation in solution [27,30]. When HMG boxes interact with DNA, the tail is displaced into solution, resulting in a complete random coil conformation. The resultant increase in the system entropy might be responsible for the enhancement in DNA bending relative to that of the tailless version. The free acidic tail could then readily bind to other structures, such as transcription factors or other proteins. In fact, interaction between the acidic tail and histones H1 and H3 was previously observed [24,25]. The sequence of events would be as follows: 1) HMGB1 interacts with the target-DNA; 2) the DNA bending favored by the acidic tail recruits other regulator/transcription factors to bind DNA; and 3) the acidic tail may interact with histones, displacing them from DNA and inducing chromatin loosening. These events might explain the role of HMGB1 in chromatin remodeling as well as its function as an architectural factor [58,59].

In summary, our studies were the first to demonstrate the role of the acidic tail of HMGB1 in protein stability and DNA bending *in vitro*. All chemical and physical denaturing agents tested were clearly shown to have a higher significant impact on the protein stability when the acidic tail was removed. Both HMGB1 and HMGB1∆C appear to have folding intermediates in acidic media, and these intermediates require further studies. The presence of the acidic tail does not contribute to the DNA-binding affinity but does significantly increase the bending angle of linear DNA upon HMGB1 binding in solution. A binding/bending model was proposed, in which the role of the acidic tail was explained in detail. 

## Materials and Methods

### Reagents

All reagents were of analytical grade. Anti-HMGB1 monoclonal antibody, ultra-pure urea, Gdn.HCl and bis-ANS were purchased from Sigma (MO, USA). SDS-PAGE standards were obtained from Bio-Rad (CA, USA). The unlabeled- and 5’-6-carboxy tetramethyl rhodamine (TAMRA)-labeled DNA sequence 5’-TACTGTATGAGCATACAGTA-3’ and its unlabeled- and carboxyfluorescein (6-FAM)-labeled complementary sequences were purchased from IDT (Iowa, USA). Unless otherwise noted, all experiments were performed in buffer containing 10 mM Tris.HCl at pH 7.5, 50 mM NaCl, 0.5 mM DTT, 0.1 mM EDTA and 5% glycerol.

### Protein expression and purification

The genes of human HMGB1 (full-length and lacking the acidic tail (ΔC)) were cloned in-frame into a pET21d-modified plasmid (Novagen, USA), which carried a 6×Histag sequence and nTev protease cleavage site in its 5’ end and was named pET21dHistev. For protein expression, the bacterial strain BL21(λDE3) + pLysS transformed with *hgmb1* gene-carrying plasmids was grown in 2 L of Luria-Bertani (LB) culture medium containing 100 μg/mL ampicillin and 34 μg/mL chloramphenicol, and gene expression was induced by the addition of 0.5 mM IPTG when the O.D._600nm_ reached 0.6-0.8. After 4 h at 37 °C and 200 rpm, cells were collected by centrifugation at 3000 g for 20 min at 4 °C. Cell pellets were resuspended in 50 mL of Buffer A (50 mM Tris.HCl at pH 8, 100 mM NaCl, 5% glycerol and 1 mM β-mercaptoethanol) containing a protease inhibitor cocktail (Sigma, MO, USA) and 1 mM PMSF. After cell lysis with 5 mg/mL lysozyme for 30 min at 4 °C, the suspension was subjected to 12 cycles of 30 s of sonication and 30 s of resting. After 30 min of centrifugation at 30,000 g and 4 °C, the pellet was discarded and the supernatant was incubated with 0.5% sodium deoxycholate for 20 min under stirring at the cold room. After 1-h centrifugation at 60,000 g at 4 °C, the pellet was discarded and the supernatant was incubated with polymin P (a 10% stock solution was previously prepared with the pH adjusted to 7.6), whose final concentration was adjusted to 0.35% (v/v), under fast stirring for 30 min. The sample was again centrifuged for 1 h at 60,000 g and 4 °C. The supernatant was then dialyzed overnight in a 3,500-MWCO dialysis bag against 4 L of buffer A. The full-length HMGB1 and HMGB1ΔC proteins were precipitated using 50, 75 and 100% (w/v) ammonium sulfate (Merck, USA). The 75 and 100% pellets were resuspended in 10 mL of Buffer A containing 500 mM NaCl and dialyzed overnight in a 3,500-MWCO dialysis bag (Spectrum Labs, USA) against 2 L of same buffer. Both proteins were purified by affinity chromatography using a 5-mL HisTrap (GE-Healthcare, USA) column and ÄKTA Purifier HPLC (GE-Healthcare, USA), according to the manufacturer’s instructions. Protein immobilization was achieved with a flow rate of 2 mL/min, and the weakly bound proteins were washed out with 10 column volumes of buffer containing 50 mM Tris.HCl at pH 8, 500 mM NaCl, 5% glycerol, 1 mM β-mercaptoethanol and 20 mM imidazole. His-tagged proteins were eluted in the same buffer but with 500 mM imidazole. For HMGB1ΔC, a further purification by ion chromatography MonoS GL 10/100 column (GE-Healthcare, USA) was necessary. The sample was diluted 5 fold and then injected onto the column using 1 mL/min flow. A continuous sodium chloride gradient from 0.1 to 1 M was used for protein elution in 4-mL aliquots. The pure proteins were visualized using 15% SDS-PAGE, followed by Coomassie blue G-250 staining (Merck, USA). HMGB1 and HMGB1ΔC were dialyzed overnight at 4 °C against 2 L of final buffer containing 10 mM Tris.HCl at pH 7.5, 50 mM NaCl, 0.5 mM DTT, 0.1 mM EDTA and 5% glycerol with a 35000 kDa membrane. The protein concentration was calculated using Bradford’s method [60].

### Western blotting

After separation in 15% SDS-PAGE, the recombinant proteins were transferred onto a PVDF membrane using 10 mM CAPS buffer (pH 11) in a Trans-blot Semi-Dry system from Bio-Rad (CA, USA), according to the manufacturer’s instructions. The membrane was blocked with 1X TBST + 5% dry milk for 2 h at 4 °C with constant stirring. Primary rabbit monoclonal anti-HMGB1 antibody (AbCam, USA) was diluted 1:1,000 and incubated overnight in the same conditions mentioned above. After 3 washes, the membrane was incubated with goat anti-rabbit secondary antibody coupled to horseradish-peroxidase (KPL) (diluted 1:4,000) for 1 h at 4 °C under constant stirring. The proteins were detected with SuperSignal West Pico Chemiluminescent Substrate (Pierce, Illinois, USA), according to the manufacturer’s instructions.

### Spectroscopic analyses

Fluorescence spectroscopy measurements were performed in a Varian Cary Eclipse spectrofluorometer (Sydney, Australia). For the Trp fluorescence, the excitation wavelength was fixed at 280 nm, and the emission spectrum was recorded from 300 to 420 nm, using slits of 5 and 10 nm in the excitation and emission paths, respectively. A 1-cm path length quartz cuvette was used. All the experiments were performed at 25 °C in the absence or presence of denaturing agents after 1-h incubation. The final protein concentration of each sample used in the measurements was quantitated by a Bradford Assay kit (Sigma, MO, USA) and adjusted to be 5 μM. Fluorescence spectra were transformed into the center of spectral mass (CM):

$$CM=\sum \upsilon_{i}\cdot F_{i}/\sum F_{i}$$ (1)

where F_i_ is the fluorescence emitted at wave number υ_i_.

The urea- or Gdn.HCl-generated protein denaturation was converted from the CM to the fraction of denatured protein (α) by the following equation:

$$\alpha ={\left[1+Q\cdot \left(CM-CM_{D}\right)/\left(CM_{N}-CM\right)\right]}^{-1}$$ (2)

where Q is the ratio between the quantum yields of the denatured and native forms, and CM_D_ and CM_N_ are the CM corresponding to the denatured and native species, respectively. The curves were fitted according to the linear extrapolation method proposed by Pace and Shaw [29]. The bis-ANS fluorescence was measured with an excitation wavelength of 360 nm, and the emission spectrum was recorded from 400 to 600 nm, using slits of 5 and 10 nm in the excitation and emission paths, respectively. The normalized spectral area (A/A_0_) was obtained by dividing the area for each bis-ANS concentration by the area value of the spectrum of this probe in buffer. For thermal denaturation experiments, the CM of the Trp emission spectra was measured over the temperature range 5-75 °C with heating at a rate of 1 °C/min and a 10-min equilibration interval between each measurement. The temperature gradient was then reversed to check whether the proteins refolded. Different pH values were obtained using a mixture of 0.1 M sodium citrate/citric acid solutions, and the spectra were acquired after a 1-h incubation period. The pH of each sample was measured after the experiments were performed to ensure their actual pH values.

DNA-protein binding was monitored by Trp quenching and the bis-ANS probe. For the Trp quenching experiments, the protein concentration was fixed at 2 μM, and 20-base pair (bp) double-stranded (ds) DNA was added until a final concentration of 2 μM was obtained. After 15 min, spectra were recorded as described above. For the bis-ANS experiments, the probe and protein concentrations were fixed at 10 and 0.5 μM, respectively. The 20-bp dsDNA concentration ranged from 0-1.2 μM, and the spectra were recorded as previously described.

### Spectropolarimetry

CD experiments were conducted in a Chirascan Circular Dichroism Spectropolarimeter (Applied Photophysics, UK) at 20 °C using a quartz cuvette with a 0.1-cm path length. Spectra from three scans from 190 to 260 nm at 30 nm/min were averaged, and the buffer baselines were subtracted from their respective sample spectra. Measurements of the molar ellipticity were calculated as follows:

$${\left[\theta \right]}_{MRW}=100\cdot \theta \cdot 10^{-3}/C_{mr}\cdot 0.1$$ (3)

where [θ]_MRW_ is the mean residue weight in degrees, C_mr_ represents the molar concentration multiplied by the number of amino acids, and 0.1 is the path length in cm. Low pH experiments were performed in 0.1 M citric acid, as previously described [61]. The final protein concentration of each sample used in the measurements was quantitated using a Bradford Assay kit and shown to be 5 μM. For thermal denaturation experiments, the ellipticity at 222 nm was followed over the temperature range of 10-80 °C with heating at a rate of 0.5 °C/min. The temperature gradient was then reversed to check whether the proteins refolded.

### Fluorescence anisotropy

Single-stranded (ss) DNA and its corresponding complementary strand at the same concentration were heated at 100 °C for 20 min in 50 mM Tris.HCl and 200 mM NaCl, and the solution was cooled down slowly to room temperature. The ds-DNA annealing was confirmed by a native 18% PAGE gel as described elsewhere [61]. For the fluorescence anisotropy, the concentration of duplex 6-FAM-labeled DNA was 50 nM. The protein concentration varied from 0 to 10 μM. The excitation and emission wavelengths were 490 and 520 nm, respectively, with a cut-off of 515 nm; 100 readings per well were collected. Samples in opaque 96-well plates from Greiner Bio-One (Kremsmünster, Austria) were read after a 10-min incubation in the dark in a SpectraMax microplate reader (CA, USA). The curves were fitted by a dose response sigmoidal function available in the Sigma Plot software program v. 10.0. The stoichiometry of binding was assessed by increasing the protein concentration with a fixed concentration of 50 nM for the fluorescent probe (FAM-DNA) and 2 μM for the non-fluorescent probe. This strategy aimed at tracking the saturation of the protein-DNA interactions. Binding was monitored as described above.

### DNA bending

For the fluorescence resonance energy transfer (FRET) analysis, 20-bp dsDNA labeled with either FAM or TAMRA at one of the 5’-end or with FAM and TAMRA at both 5’-ends was used at 50 nM. HMGB1 and HMGB1ΔC were diluted to 5 μM in a reaction volume of 100 μL. The reactions were read in a SpectraMax M5 microplate reader with an excitation wavelength of 490 nm for the FAM and FAM-TAMRA probes and 540 nm for the FAM probe only. The emission spectra were collected at 520 nm for the FAM probe and 580 nm for the TAMRA and FAM-TAMRA probes. The efficiency of energy transfer E of a donor-acceptor pair at distance R was calculated as previously described [38]:

$$E=R_{0}^{6}/\left(R_{0}^{6}+R^{6}\right)$$ (4)

where R_0_ for FAM-TAMRA probes, which represents the distance for 50% energy transfer efficiency, is 50 Å [62]. The calculations included corrections for possible effects of protein binding on the probes and interference between FAM and TAMRA. The DNA bending angle was correlated with the probe’s distance by the two-kinked model of HMGB1 bending [40,41,50].

## References

[1] Goodwin GH, Sanders C, Johns EW (1973) A new group of chromatin-associated proteins with a high content of acidic and basic amino acids. Eur J Biochem 38: 14-19. doi:10.1111/j.1432-1033.1973.tb03026.x. PubMed: 4774120. 4774120

[2] Weir HM, Kraulis PJ, Hill CS, Raine AR, Laue ED et al. (1993) Structure of the HMG box motif in the B-domain of HMG1. EMBO J 12: 1311-1319. PubMed: 8467791. 8467791 10.1002/j.1460-2075.1993.tb05776.x PMC413342

[3] Hardman CH, Broadhurst RW, Raine ARC, Grasser KD, Thomas JO et al. (1995) Structure of the A-domain of HMG1 and its interaction with DNA as studied by heteronuclear three- and four-dimensional NMR spectroscopy. Biochemistry 34: 16596-16607. doi:10.1021/bi00051a007. PubMed: 8527432. 8527432

[4] Thomas JO, Travers AA (2001) HMG1 and 2, and related 'architectural' DNA-binding proteins. Trends Biochem Sci 26: 167-174. doi:10.1016/S0968-0004(01)01801-1. PubMed: 11246022. 11246022

[5] Thomas JO (2001) HMG 1 and 2: architectural DNA-binding proteins. Biochem Soc Trans 29: 395-401. doi:10.1042/BST0290395. PubMed: 11497996. 11497996

[6] Gerlitz G, Hock R, Ueda T, Bustin M (2009) The dynamics of HMG protein-chromatin interactions in living cells. Biochem Cell Biol 87: 127-137. doi:10.1139/O08-110. PubMed: 19234529. 19234529 PMC3459335

[7] Hamada H, Bustin M (1985) Hierarchy of binding sites for chromosomal proteins HMG 1 and 2 in supercoiled deoxyribonucleic acid. Biochemistry 24: 1428-1433. doi:10.1021/bi00327a022. PubMed: 2985113. 2985113

[8] Bianchi ME, Beltrame M, Paonessa G (1989) Specific recognition of cruciform DNA by nuclear protein HMG1. Science 243: 1056-1059. doi:10.1126/science.2922595. PubMed: 2922595. 2922595

[9] Gaillard C, Strauss F (2000) High affinity binding of proteins HMG1 and HMG2 to semicatenated DNA loops. BMC Mol Biol 1: 1. doi:10.1186/1471-2199-1-1. PubMed: 11041984. 11041984 PMC29088

[10] Webb M, Payet D, Lee KB, Travers AA, Thomas JO (2001) Structural requirements for cooperative binding of HMG1 to DNA minicircles. J Mol Biol 309: 79-88. doi:10.1006/jmbi.2001.4667. PubMed: 11491303. 11491303

[11] Sessa L, Bianchi ME (2007) The evolution of High Mobility Group Box (HMGB) chromatin proteins in multicellular animals. Gene 387: 133-140. doi:10.1016/j.gene.2006.08.034. PubMed: 17156942. 17156942

[12] Müller S, Ronfani L, Bianchi ME (2004) Regulated expression and subcellular localization of HMGB1, a chromatin protein with a cytokine function. J Intern Med 255: 332-343. doi:10.1111/j.1365-2796.2003.01296.x. PubMed: 14871457. 14871457

[13] Scaffidi P, Misteli T, Bianchi ME (2002) Release of chromatin protein HMGB1 by necrotic cells triggers inflammation. Nature 418: 191-195. doi:10.1038/nature00858. PubMed: 12110890. 12110890

[14] Phair RD, Scaffidi P, Elbi C, Vecerová J, Dey A et al. (2004) Global nature of dynamic protein-chromatin interactions in vivo: three-dimensional genome scanning and dynamic interaction networks of chromatin proteins. Mol Cell Biol 24: 6393-6402. doi:10.1128/MCB.24.14.6393-6402.2004. PubMed: 15226439. 15226439 PMC434243

[15] Heyduk E, Heyduk T, Claus P, Wiśniewski JR (1997) Conformational changes of DNA induced by binding of *Chironomus* high mobility group protein 1a (cHMG1a) - Regions flanking an HMG1 box domain do not influence the bend angle of the DNA. J Biol Chem 272: 19763-19770. doi:10.1074/jbc.272.32.19763. PubMed: 9242635. 9242635

[16] Lorenz M, Hillisch A, Payet D, Buttinelli M, Travers A et al. (1999) DNA bending induced by high mobility group proteins studied by fluorescence resonance energy transfer. Biochemistry 38: 12150-12158. doi:10.1021/bi990459 + PubMed : 10508419 10508419

[17] McCauley MJ, Zimmerman J, Maher LJ III, Williams MC (2007) HMGB binding to DNA: single and double box motifs. J Mol Biol 374: 993-1004. doi:10.1016/j.jmb.2007.09.073. PubMed: 17964600. 17964600 PMC2117627

[18] Zhang J, McCauley MJ, Maher LJ III, Williams MC, Israeloff NE (2009) Mechanism of DNA flexibility enhancement by HMGB proteins. Nucleic Acids Res 37: 1107-1114. PubMed: 19129233. 19129233 10.1093/nar/gkn1011 PMC2651801

[19] Stros M (1998) DNA bending by the chromosomal protein HMG1 and its high mobility group box domains - Effect of flanking sequences. J Biol Chem 273: 10355-10361. PubMed: 9553091. 9553091

[20] Ellwood KB, Yen YM, Johnson RC, Carey M (2000) Mechanism for specificity by HMG-1 in enhanceosome assembly. Mol Cell Biol 20: 4359-4370. doi:10.1128/MCB.20.12.4359-4370.2000. PubMed: 10825199. 10825199 PMC85803

[21] Sheflin LG, Fucile NW, Spaulding SW (1993) The specific interactions of HMG 1 and 2 with negatively supercoiled DNA are modulated by their acidic C-terminal domains and involve cysteine residues in their HMG 1/2 boxes. Biochemistry 32: 3238-3248. doi:10.1021/bi00064a005. PubMed: 8461290. 8461290

[22] Stros M, Stokrová J, Thomas JO (1994) DNA looping by the HMG-box domains of HMG1 and modulation of DNA binding by the acidic C-terminal domain. Nucleic Acids Res 22: 1044-1051. doi:10.1093/nar/22.6.1044. PubMed: 8152909. 8152909 PMC307928

[23] Payet D, Travers A (1997) The acidic tail of the high mobility group protein HMG-D modulates the structural selectivity of DNA binding. J Mol Biol 266: 66-75. doi:10.1006/jmbi.1996.0782. PubMed: 9054971. 9054971

[24] Ueda T, Chou H, Kawase T, Shirakawa H, Yoshida M (2004) Acidic C-tail of HMGB1 is required for its target binding to nucleosome linker DNA and transcription stimulation. Biochemistry 43: 9901-9908. doi:10.1021/bi035975l. PubMed: 15274644. 15274644

[25] Cato L, Stott K, Watson M, Thomas JO (2008) The interaction of HMGB1 and linker histones occurs through their acidic and basic tails. J Mol Biol 384: 1262-1272. doi:10.1016/j.jmb.2008.10.001. PubMed: 18948112. 18948112

[26] Knapp S, Müller S, Digilio G, Bonaldi T, Bianchi ME et al. (2004) The long acidic tail of high mobility group box 1 (HMGB1) protein forms an extended and flexible structure that interacts with specific residues within and between the HMG boxes. Biochemistry 43: 11992-11997. doi:10.1021/bi049364k. PubMed: 15379539. 15379539

[27] Watson M, Stott K, Thomas JO (2007) Mapping intramolecular interactions between domains in HMGB1 using a tail-truncation approach. J Mol Biol 374: 1286-1297. doi:10.1016/j.jmb.2007.09.075. PubMed: 17988686. 17988686

[28] Mohana-Borges R, Pacheco AB, Sousa FJ, Foguel D, Almeida DF et al. (2000) LexA repressor forms stable dimers in solution. The role of specific DNA in tightening protein-protein interactions. J Biol Chem 275: 4708-4712. doi:10.1074/jbc.275.7.4708. PubMed: 10671501. 10671501

[29] Pace CN, Shaw KL (2000) Linear extrapolation method of analyzing solvent denaturation curves. Proteins Suppl 4: 1-7. PubMed: 11013396. 10.1002/1097-0134(2000)41:4+<1::aid-prot10>3.3.co;2-u 11013396

[30] Stott K, Watson M, Howe FS, Grossmann JG, Thomas JO (2010) Tail-mediated collapse of HMGB1 is dynamic and occurs via differential binding of the acidic tail to the A and B domains. J Mol Biol 403: 706-722. doi:10.1016/j.jmb.2010.07.045. PubMed: 20691192. 20691192

[31] Takashi R, Tonomura Y, Morales MF (1977) 4,4'-Bis (1-anilinonaphthalene 8-sulfonate) (bis-ANS): a new probe of the active site of myosin. Proc Natl Acad Sci U S A 74: 2334-2338. doi:10.1073/pnas.74.6.2334. PubMed: 267928. 267928 PMC432165

[32] Elenkov I, Pelovsky P, Ugrinova I, Takahashi M, Pasheva E (2011) The DNA binding and bending activities of truncated tail-less HMGB1 protein are differentially affected by Lys-2 and Lys-81 residues and their acetylation. Int J Biol Sci 7: 691-699. PubMed: 21647302. 21647302 10.7150/ijbs.7.691 PMC3107488

[33] Zimmerman J, Maher LJ III (2008) Transient HMGB protein interactions with B-DNA duplexes and complexes. Biochem Biophys Res Commun 371: 79-84. doi:10.1016/j.bbrc.2008.04.024. PubMed: 18413230. 18413230 PMC2408743

[34] Wang Q, Zeng M, Wang W, Tang J (2007) The HMGB1 acidic tail regulates HMGB1 DNA binding specificity by a unique mechanism. Biochem Biophys Res Commun 360: 14-19. doi:10.1016/j.bbrc.2007.05.130. PubMed: 17585880. 17585880

[35] Carpenter ML, Oliver AW, Kneale GG (2001) Analysis of DNA-protein interactions by intrinsic fluorescence. In: Moss T DNA-protein Interactions: principles and protocols. New Jersey: Human Press Inc. pp. 491-502. 10.1385/1-59259-208-2:491 11357608

[36] Weinberg RL, Veprintsev DB, Fersht AR (2004) Cooperative binding of tetrameric p53 to DNA. J Mol Biol 341: 1145-1159. doi:10.1016/j.jmb.2004.06.071. PubMed: 15321712. 15321712

[37] Polyanichko AM, Chikhirzhina EV, Skvortsov AN, Kostyleva EI, Colson P et al. (2002) The HMG1 ta. (p. i )le. J Biomol Struct Dyn 19: 1053-1062 10.1080/07391102.2002.10506808 12023807

[38] Kugel JF (2008) Using FRET to measure the angle at which a protein bends DNA: TBP binding a TATA box as a model system. Biochem Mol Biol Educ 36: 341-346. doi:10.1002/bmb.20202. PubMed: 21591217. 21591217

[39] Dragan AI, Privalov PL (2008) Use of fluorescence resonance energy transfer (FRET) in studying protein-induced DNA bending. Methods Enzymol 450: 185-199. doi:10.1016/S0076-6879(08)03409-5. PubMed: 19152861. 19152861

[40] Masters KM, Parkhurst KM, Daugherty MA, Parkhurst LJ (2003) Native human TATA-binding protein simultaneously binds and bends promoter DNA without a slow isomerization step or TFIIB requirement. J Biol Chem 278: 31685-31690. doi:10.1074/jbc.M305201200. PubMed: 12791683. 12791683

[41] Hieb AR, Halsey WA, Betterton MD, Perkins TT, Kugel JF et al. (2007) TFIIA changes the conformation of the DNA in TBP/TATA complexes and increases their kinetic stability. J Mol Biol 372: 619-632. doi:10.1016/j.jmb.2007.06.061. PubMed: 17681538. 17681538

[42] Allonso D, Belgrano FS, Calzada N, Guzmán MG, Vázquez S et al. (2012) Elevated serum levels of high mobility group box 1 (HMGB1) protein in dengue-infected patients are associated with disease symptoms and secondary infection. J Clin Virol 55: 214-219. doi:10.1016/j.jcv.2012.07.010. PubMed: 22884669. 22884669

[43] Trøseid M, Nowak P, Nyström J, Lindkvist A, Abdurahman S et al. (2010) Elevated plasma levels of lipopolysaccharide and high mobility group box-1 protein are associated with high viral load in HIV-1 infection: reduction by 2-year antiretroviral therapy. AIDS 24: 1733-1737. doi:10.1097/QAD.0b013e32833b254d. PubMed: 20502315. 20502315

[44] Tang D, Kang R, Zeh HJ III, Lotze MT (2010) High-mobility group box 1 and cancer. Biochim Biophys Acta 1799: 131-140. doi:10.1016/j.bbagrm.2009.11.014. PubMed: 20123075. 20123075 PMC2818552

[45] Myers JK, Pace CN, Scholtz JM (1995) Denaturant m values and heat capacity changes: relation to changes in accessible surface areas of protein unfolding. Protein Sci 4: 2138-2148. doi:10.1002/pro.5560041020. PubMed: 8535251. 8535251 PMC2142997

[46] Anderson DE, Becktel WJ, Dahlquist FW (1990) pH-induced denaturation of proteins: A single salt bridge contributes 3-5 kcal/mol to the free energy of folding of T4 lysozyme. Biochemistry 29: 2403-2408. doi:10.1021/bi00461a025. PubMed: 2337607. 2337607

[47] Dragan AI, Klass J, Read C, Churchill ME, Crane-Robinson C et al. (2003) DNA binding of a non-sequence-specific HMG-D protein is entropy driven with a substantial non-electrostatic contribution. J Mol Biol 331: 795-813. doi:10.1016/S0022-2836(03)00785-X. PubMed: 12909011. 12909011

[48] Stros M (2010) HMGB proteins: interactions with DNA and chromatin. Biochim Biophys Acta 1799: 101-113. doi:10.1016/j.bbagrm.2009.09.008. PubMed: 20123072. 20123072

[49] Pasheva E, Sarov M, Bidjekov K, Ugrinova I, Sarg B et al. (2004) In vitro acetylation of HMGB-1 and -2 proteins by CBP: the role of the acidic tail. Biochemistry 43: 2935-2940. doi:10.1021/bi035615y. PubMed: 15005629. 15005629

[50] Wu J, Parkhurst KM, Powell RM, Brenowitz M, Parkhurst LJ (2001) DNA bends in TATA-binding protein-TATA complexes in solution are DNA sequence-dependent. J Biol Chem 276: 14614-14622. doi:10.1074/jbc.M004402200. PubMed: 11278276. 11278276

[51] Kim Y, Geiger JH, Hahn S, Sigler PB (1993) Crystal structure of a yeast TBP/TATA-box complex. Nature 365: 512-520. doi:10.1038/365512a0. PubMed: 8413604. 8413604

[52] Werner MH, Huth JR, Gronenborn AM, Clore GM (1995) Molecular basis of human 46X,Y sex reversal revealed from the three-dimensional solution structure of the human SRY-DNA complex. Cell 81: 705-714. doi:10.1016/0092-8674(95)90532-4. PubMed: 7774012. 7774012

[53] Rice PA, Yang SW, Mizuuchi K, Nash HA (1996) Crystal structure of an IHF-DNA complex: A protein-induced DNA u-turn. Cell 87: 1295-1306. doi:10.1016/S0092-8674(00)81824-3. PubMed: 8980235. 8980235

[54] Masse JE, Wong B, Yen YM, Allain FH, Johnson RC et al. (2002) The S. cerevisiae architectural HMGB protein NHP6A complexed with DNA: DNA and protein conformational changes upon binding. J Mol Biol 323: 263-284. doi:10.1016/S0022-2836(02)00938-5. PubMed: 12381320. 12381320

[55] McCauley M, Hardwidge PR, Maher LJ III, Williams MC (2005) Dual binding modes for an HMG domain from human HMGB2 on DNA. Biophys J 89: 353-364. doi:10.1529/biophysj.104.052068. PubMed: 15833996. 15833996 PMC1366535

[56] Aizawa S, Nishino H, Saito K, Kimura K, Shirakawa H et al. (1994) Stimulation of transcription in cultured cells by high mobility group protein 1: Essential role of the acidic carboxyl-terminal region. Biochemistry 33: 14690-14695. doi:10.1021/bi00253a006. PubMed: 7993897. 7993897

[57] Bonaldi T, Längst G, Strohner R, Becker PB, Bianchi ME (2002) The DNA chaperone HMGB1 facilitates ACF/CHRAC-dependent nucleosome sliding. EMBO J 21: 6865-6873. doi:10.1093/emboj/cdf692. PubMed: 12486007. 12486007 PMC139112

[58] Travers AA (2003) Priming the nucleosome: a role for HMGB proteins? EMBO Rep 4: 131-136. doi:10.1038/sj.embor.embor741. PubMed: 12612600. 12612600 PMC1315838

[59] Müller S, Scaffidi P, Degryse B, Bonaldi T, Ronfani L et al. (2001) The double life of HMGB1 chromatin protein: architectural factor and extracellular signal. EMBO J 20: 4337-4340. doi:10.1093/emboj/20.16.4337. PubMed: 11500360. 11500360 PMC125571

[60] Bradford MM (1976) A rapid and sensitive method for the quantitation of microgram quantities of protein utilizing the principle of protein-dye binding. Anal Biochem 72: 248-254. doi:10.1016/0003-2697(76)90527-3. PubMed: 942051. 942051

[61] Sousa FJ, Lima LM, Pacheco AB, Oliveira CL, Torriani I et al. (2006) Tetramerization of the LexA Repressor in Solution: Implications for Gene Regulation of the E.coli SOS System at Acidic pH. J Mol Biol 359: 1059-1074. doi:10.1016/j.jmb.2006.03.069. PubMed: 16701697. 16701697

[62] Dragan AI, Read CM, Makeyeva EN, Milgotina EI, Churchill ME et al. (2004) DNA binding and bending by HMG boxes: energetic determinants of specificity. J Mol Biol 343: 371-393. doi:10.1016/j.jmb.2004.08.035. PubMed: 15451667. 15451667

